# Dipole pattern of meridional atmospheric internal energy transport across the Arctic gate

**DOI:** 10.1038/s41598-022-06371-9

**Published:** 2022-02-11

**Authors:** Mikhail M. Latonin, Leonid P. Bobylev, Igor L. Bashmachnikov, Richard Davy

**Affiliations:** 1 Saint Petersburg State University, Universitetskaya Emb. 7–9, 199034 Saint Petersburg, Russia; 2 Nansen International Environmental and Remote Sensing Centre, 14th Line 7, Office 49, Vasilievsky Island, 199034 Saint Petersburg, Russia; 3 Nansen Environmental and Remote Sensing Center, and Bjerknes Center for Climate Research, Jahnebakken 3, N-5007 Bergen, Norway

**Keywords:** Atmospheric science, Climate change, Atmospheric dynamics

## Abstract

High-latitude atmospheric meridional energy transport plays a fundamental role in the Arctic climate system. However, despite numerous studies, there are no established clear regional features of the atmospheric energy transport components from a large-scale perspective. This study aims at investigating the internal energy and its instantaneous sensible and latent heat transports in the troposphere across the Arctic gate at 70°N using the high-resolution climate reanalysis ERA5. We have done a regional analysis of the time series of heat fluxes across the zonal section and found by decomposing them into empirical orthogonal functions that they have opposing features for the Eastern and Western Hemispheres. In particular, the sensible heat transport dominates in the Western Hemisphere, whereas the latent heat transport dominates in the Eastern Hemisphere. Moreover, we detected the existence of an anti-phase dipole pattern for each of these components in the entire troposphere, which is robust because it continued during the entire studied period 1950–2019. The hemispheric net fluxes indicate that the Arctic gains internal energy mostly due to the latent heat transport.

## Introduction

Atmospheric meridional energy transport (AMET) can be decomposed into internal energy transport (sensible and latent heat fluxes), potential, and kinetic energy transports. The latter component is often disregarded due to its negligible contribution^1,2^. The remaining components are often referred to as the moist static energy transport^3,4^. In turn, each of them consists of transient eddies, stationary eddies, mean meridional circulation and net mass flow^3,5^. The latter term must tend to zero at monthly and longer time scales to fulfill the mass conservation law^6^. However, there is no conventional way for computing AMET. One of the reasons for that is a desire to obtain a value with a specific unit of measurement, such as corresponding to the specific humidity for moisture transport, by applying chosen normalisations and weights^5,7–9^.

Despite some discrepancies in the methodologies, important pathways and sources of AMET relevant for the Arctic have previously been identified. These are mostly the oceanic areas in the North Atlantic and North Pacific during the cold season^10,11^ and continental areas in Eurasia and North America during the warm season, especially for the latent heat component of the internal energy^12,13^. The AMET is most often calculated at the 70°N parallel, because this is a region of strong influence of the polar vortex^14^, and Bjerknes compensation is strongest at this latitude^15^.

Among the AMET and its internal energy components, the latent heat flux into the Arctic is of the highest interest as it initiates a regional feedback by increasing the downward longwave radiation, and has a further potential of melting the Arctic sea ice^16^. For instance, Zhuo and Jiang^17^ demonstrated that 60% of winter sea ice decline in the Bering Sea is explained by the near-surface southeasterly winds and 40% by a subsequent change in the downward longwave radiation. The summertime moisture transport was also found to strongly shape the sea ice area in the Arctic Pacific sector^7^. More globally, the importance of AMET in the current Arctic warming was documented in numerical experiments^4^ and in a modelling of an energy budget for possible future Arctic warming^18^.

The main purpose of this research is to investigate whether a large-scale regionalization of the atmospheric internal energy components is possible based on the time-altitude variability across the parallel at 70°N. In previous studies, the primary focus has been on the relatively small-scale regional features of AMET or its individual components.

In this study, we discovered a novel large-scale division of monthly internal energy transport at the latitude 70°N based on the analysis of empirical orthogonal functions applied to the longitude–height sections of the sensible and latent heat fluxes. According to radiosonde observations, these two fluxes are the major components and constitute more than 70% in the net energy transport into the Arctic^6^. The potential energy flux is not considered in this study.

## Results

### Mean meridional component of wind velocity in the atmospheric layers around 70°N

**Figure 1:**
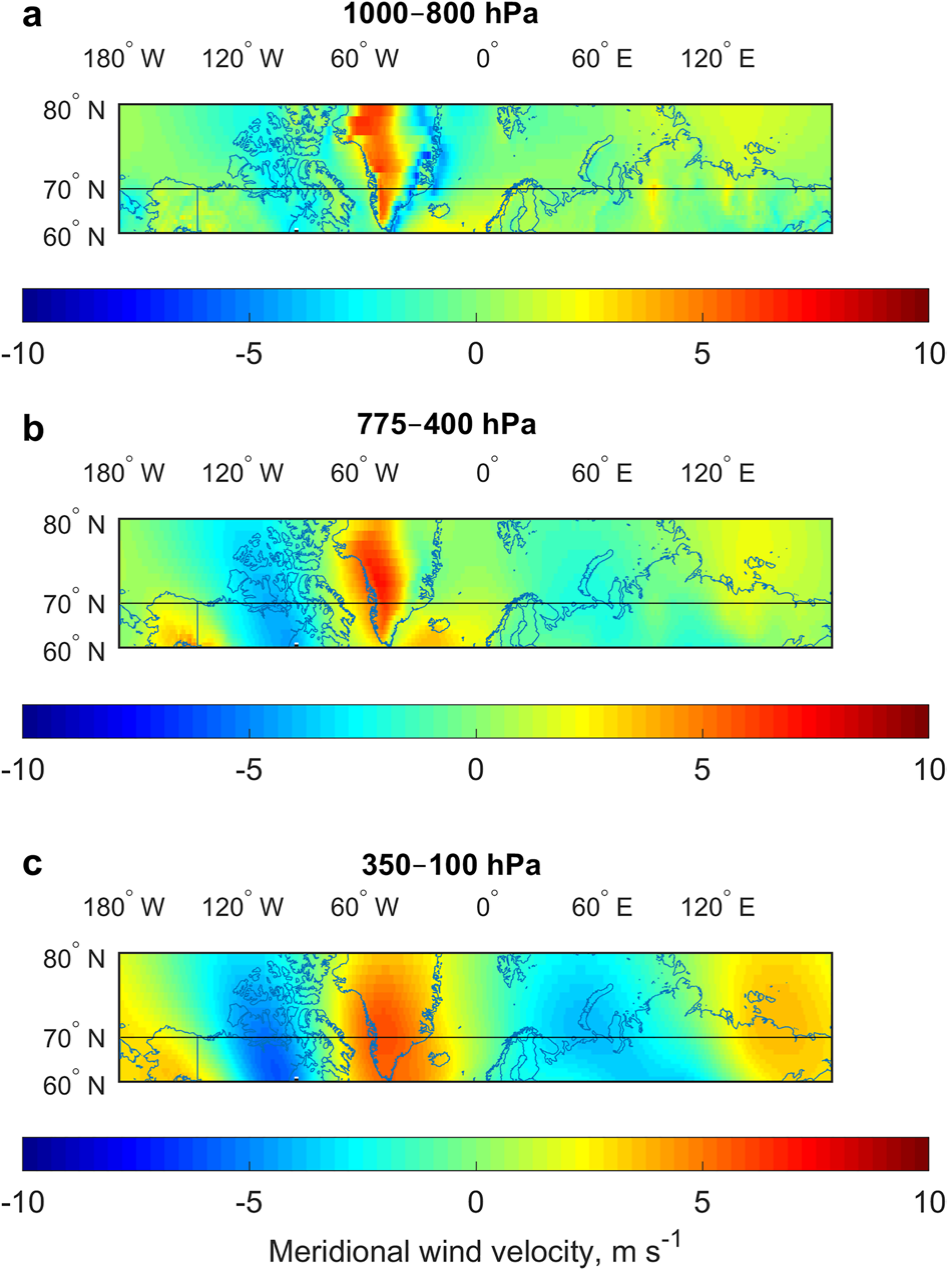
Mean meridional component of wind velocity (m s^−1^) for the region 60°–80°N in the different atmospheric layers averaged for 1950–2019. (**a**) 1000–800 hPa. (**b**) 775–400 hPa. (**c**) 350–100 hPa. Positive values indicate northward direction. The reference lines are drawn at the 70°N. Mercator map projection is used. The Matrix Laboratory (MATLAB) with version R2017b (https://www.mathworks.com/) was used to generate this figure.

For any component of AMET, an essential variable is the northward wind velocity. Therefore, we first investigated the wind fields around the parallel 70°N (Fig. 1).

The three chosen layers represent the lower troposphere with a direct interaction with the Earth’s surface, the middle troposphere with a steering flow and the upper troposphere, where the main jet streams are located. The calculation of AMET through the latitude 70°N is consistent with the meridional wind fields, as this parallel crosses the large areas of increased wind velocity near their centres. The results show that the meridional winds of both directions are the strongest over North America and Greenland. In the lower troposphere, the winds of both directions are weak over Eurasia, and there is a steady northward direction (Fig. 1a). The most clear picture of the alternating wind directions arises in the upper troposphere (Fig. 1c).

### Analysis of empirical orthogonal functions for the time series of sensible and latent heat transport components and the resulting large-scale regional division

**Figure 2:**
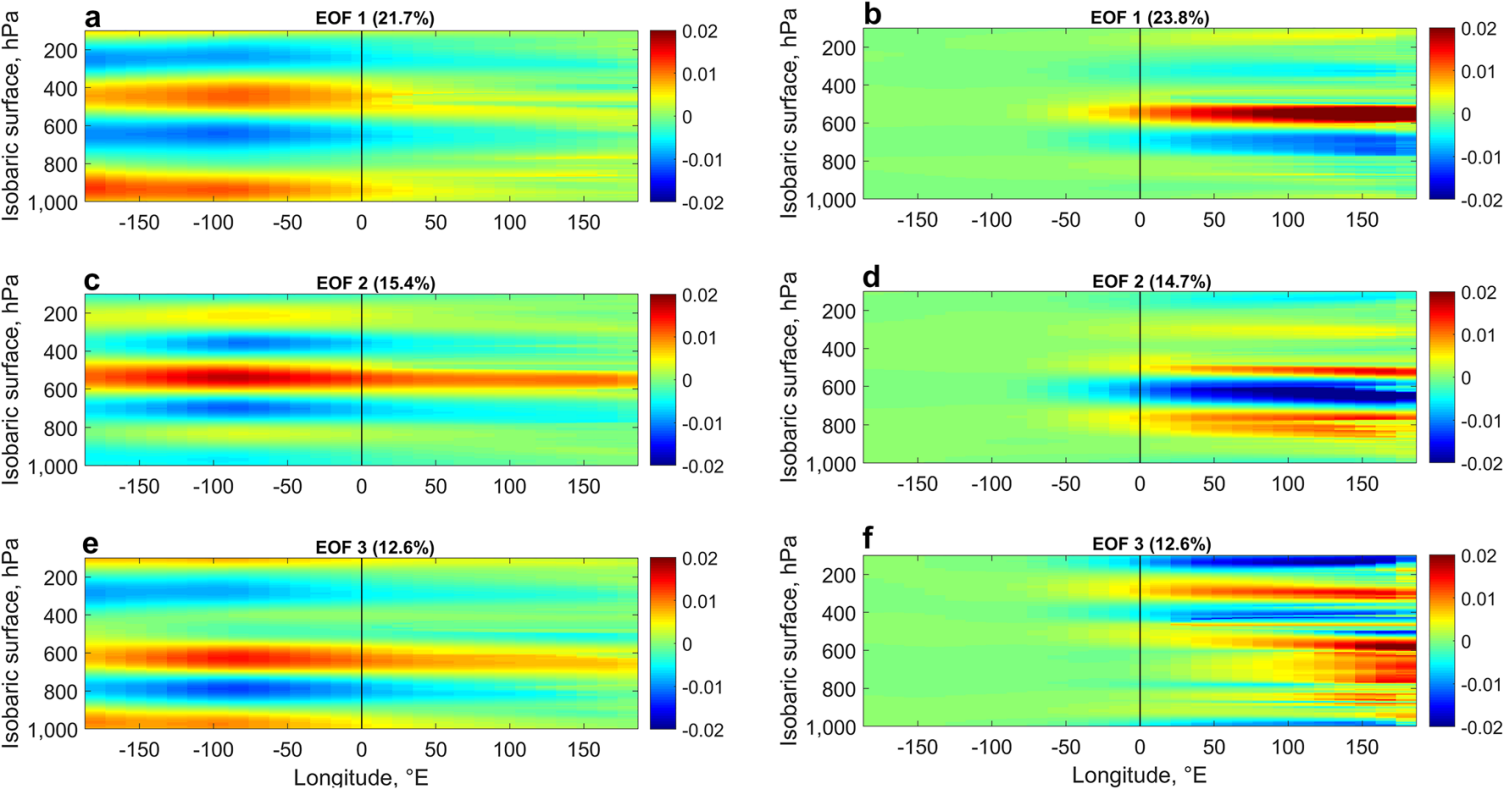
Empirical orthogonal functions (EOFs) of the sensible (left) and latent (right) heat transport components (SHT and LHT). (**a**) First EOF for the SHT (*21.7%*). (**b**) First EOF for the LHT (*23.8%*). (**c**) Second EOF for the SHT (*15.4%*). (**d**) Second EOF for the LHT (*14.7%*). (**e**) Third EOF for the SHT (*12.6%*). (**f**) Third EOF for the LHT (*12.6%*). The reference lines are drawn at the prime meridian 0°. The values in the parentheses are the fractions of variance explained by each mode. The Matrix Laboratory (MATLAB) with version R2017b (https://www.mathworks.com/) was used to generate this figure.

The time series of sensible and latent heat transports across 70°N (Eqs. 1 and 2) were decomposed into empirical orthogonal functions (EOFs) (Fig. 2).

The leading modes of variability are almost exactly divided into the Eastern and Western Hemispheres: variability of the sensible heat transport dominates in the Western Hemisphere, whereas that of the latent heat transport dominates in the Eastern Hemisphere. This division is more pronounced for the latent heat transport. The three EOFs in the figure explain about 50% of the variability for each heat transport component. The corresponding smoothed principal component time series are shown in Supplementary Fig. S1. The correlation coefficients between the unsmoothed principal component time series for the first three EOFs, representing sensible and latent heat transports, are 0.17, − 0.13, and 0.03, respectively. They are very low, which confirms significant differences in the time-altitude variability between the sensible and latent heat transports. The hemispheric pattern in the modes of variability with a similar nature of the time-altitude variability of the heat transport components is preserved if additional EOFs are added. For the sensible heat transport, the first eight EOFs explain 81% of the variance, and the first eight EOFs for the latent heat transport explain 79% of the variance (Fig. 2 and Supplementary Fig. S2). These results confirm the robustness of the identified hemispheric division for the components of heat transport. The possible physical reasons are addressed further.

### Anti-phase pattern in the sensible and latent heat transports between the Eastern and Western Hemispheres

**Figure 3:**
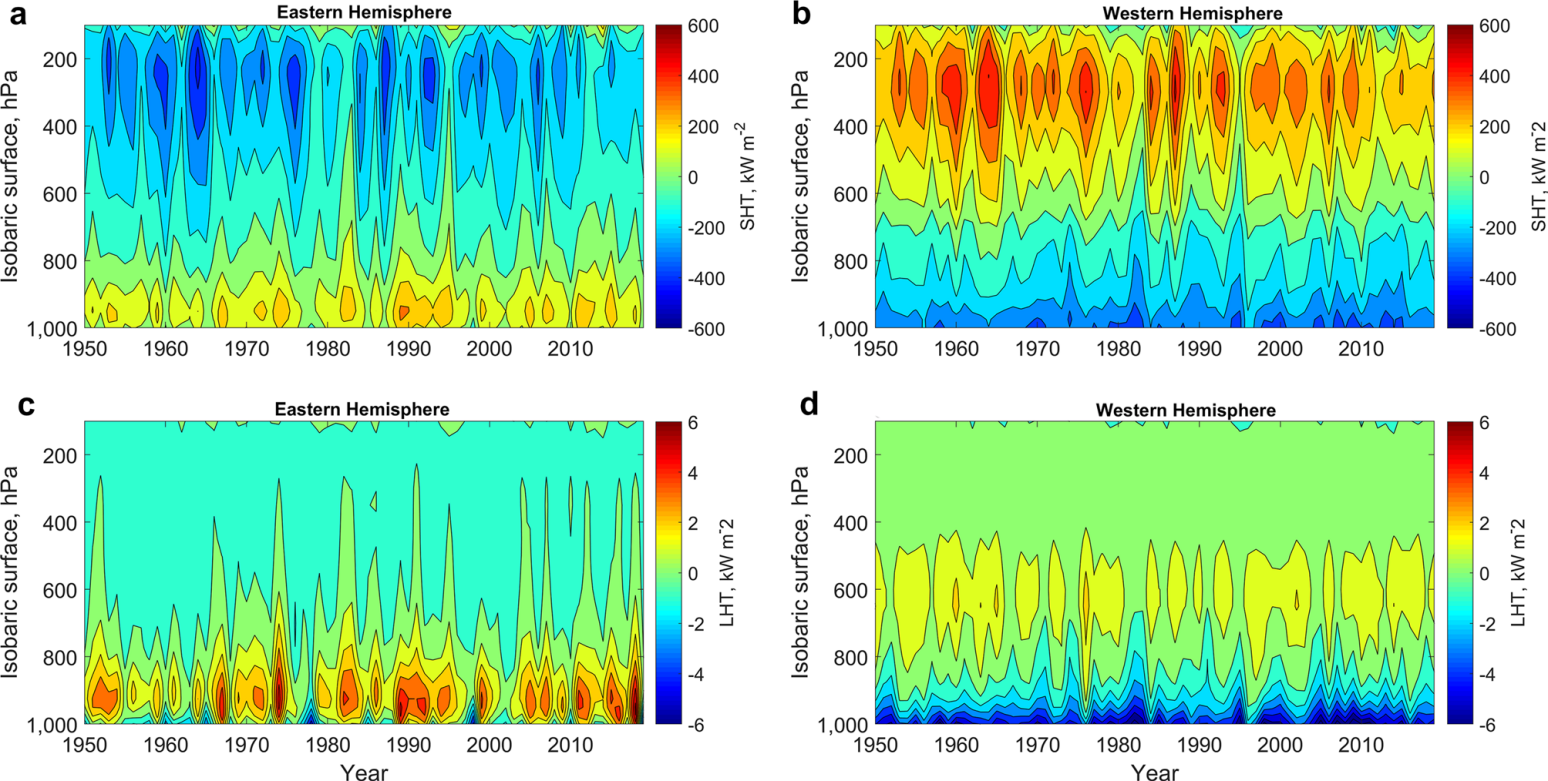
Vertical cross-section of heat transport components (kW m^−2^), their interannual variability and the anti-phase pattern between the hemispheres. (**a**) Annual average sensible heat transport in the Eastern Hemisphere (0°–179.75°E). (**b**) Annual average sensible heat transport in the Western Hemisphere (180°–0.25°W). (**c**) Annual average latent heat transport in the Eastern Hemisphere (0°–179.75°E). (**d**) Annual average latent heat transport in the Western Hemisphere (180°–0.25°W). The positive values indicate northward heat transport. The Matrix Laboratory (MATLAB) with version R2017b (https://www.mathworks.com/) was used to generate this figure.

In order to examine the vertical structure of sensible and latent heat transports, they were longitudinally averaged for the Eastern and Western Hemispheres. The noisy monthly time series were averaged to obtain annual means (Fig. 3a–d).

These time-altitude diagrams demonstrate the prevalence of sensible and latent heat transports into the Arctic in the lower troposphere (1000–800 hPa) and from the Arctic in the middle and upper troposphere for the Eastern Hemisphere. For the Western Hemisphere, the vertical distribution of the heat fluxes is opposite, which indicates a dipole anti-phase pattern between the hemispheres. According to the concept of the polar circulation cell^19^, heat transport should be directed into the Arctic in the upper troposphere and from the Arctic in the lower troposphere, which is true for the Western Hemisphere, but completely different in the Eastern Hemisphere. However, the anti-phase pattern is more stable for the sensible heat transport, because for the latent heat transport, there are layers with the same direction of fluxes in both hemispheres. For instance, these features are seen around 700–800 hPa (Fig. 3c,d).

The vertical cross-sections (Fig. 3a–d) show visually notable differences in the variability between the hemispheres, especially for the latent heat transport. Indeed, the interannual standard deviation averaged over the height of the troposphere (1000–100 hPa) is 0.61 kW m^−2^ for the latent heat transport in the Eastern Hemisphere and only 0.45 kW m^−2^ in the Western Hemisphere. This means the interannual variability of latent heat transport is 1.36 times higher in the Eastern Hemisphere than in the Western Hemisphere. For the sensible heat transport, the interannual variability is comparable, being 68.45 kW m^−2^ in the Western Hemisphere and 71.53 kW m^−2^ in the Eastern Hemisphere, which is only 1.05 times higher. However, the temporal mean of the standard deviations over the isobaric surfaces reveals the dominance of sensible heat transport in the Western Hemisphere because it is 192.7 kW m^−2^ here versus 130.97 kW m^−2^ in the Eastern Hemisphere. This means the variability of sensible heat transport over the isobaric surfaces is 1.47 times higher in the Western Hemisphere than in the Eastern Hemisphere, which is also supported by a stronger vertical colour gradient in Fig. 3b than in Fig. 3a. The same estimates for the latent heat transport give a value of 1.35 kW m^−2^ for the Western Hemisphere and 1 kW m^−2^ for the Eastern Hemisphere. This means the variability of latent heat transport over the isobaric surfaces is 1.35 times higher in the Western Hemisphere than in the Eastern Hemisphere.

Thus, the hemispheric division based on the modes of variability in Fig. 2 corresponds to the highest ratios in the standard deviations (1.36 and 1.47). Whether the variability with time or height (isobaric surfaces) dominates the overall variability in latent heat transport and sensible heat transport depends upon the hemisphere. The clearer division into the Eastern and Western Hemispheres in Fig. 2 for the latent heat transport might be due to a higher overall importance of interannual standard deviations at different isobaric surfaces compared to standard deviations over isobaric surfaces for each year.

### Fully integrated sensible and latent heat transports in the lower and entire troposphere and their net values through the Arctic gate

**Figure 4:**
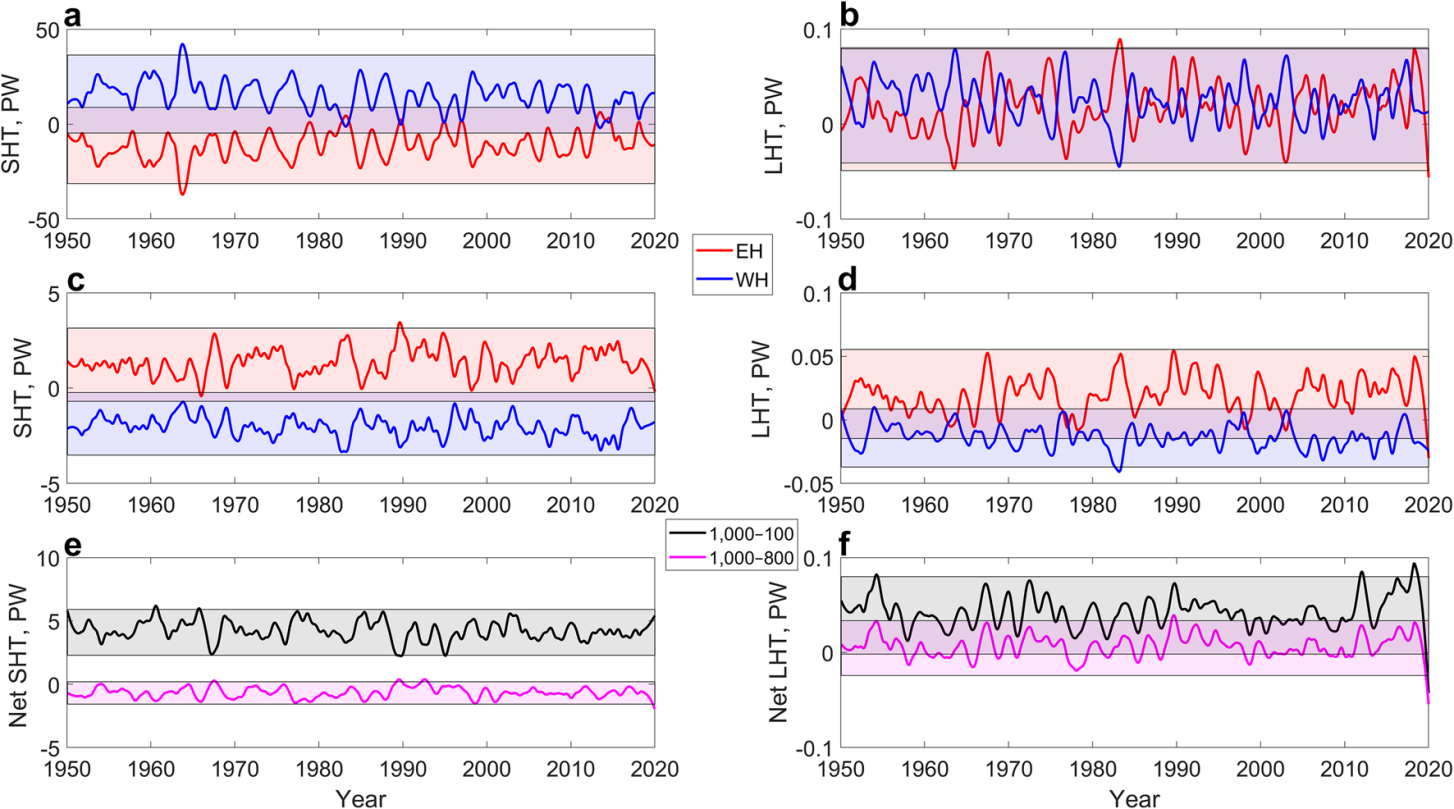
Smoothed time series of integral sensible and latent heat transport (SHT and LHT) components (Petawatt (PW)) in the lower and entire troposphere for the Eastern and Western Hemispheres (EH and WH) and the net fluxes. (**a**) 1000–100 hPa integral SHT in the EH and WH. (**b**) 1000–100 hPa integral LHT in the EH and WH. (**c**) 1000–800 hPa integral SHT in the EH and WH. (**d**) 1000–800 hPa integral LHT in the EH and WH. (**e**) Net fluxes of SHT (EH + WH). (**f**) Net fluxes of LHT (EH + WH). The lighter shaded areas indicate the interquartile ranges (differences between 75th and 25th percentiles) for the unsmoothed monthly time series, and the darker shaded areas show the intersections of the interquartile ranges for each pair of the time series. The loess smoothing was performed with a span of 5% of the values. The Matrix Laboratory (MATLAB) with version R2017b (https://www.mathworks.com/) was used to generate this figure.

The monthly integral sensible and latent heat transports in the lower troposphere (1000–800 hPa) and the entire troposphere (1000–100 hPa) were studied (Fig. 4). The layers were identified based on the previous results on the mean directions of heat fluxes (Fig. 3).

The loess smoothing was applied to demonstrate the anti-phase pattern between the hemispheres (Fig. 4a–d) and the sign of the net fluxes (Fig. 4e,f). For the sensible heat transport in the entire troposphere (Fig. 4a,e), heat transport is mostly directed into the Arctic due to a stronger mean heat influx in the Western Hemisphere than heat outflux in the Eastern Hemisphere. The correlation coefficients between the heat transports in the hemispheres for the unsmoothed and smoothed time series used in the Fig. 4a are − 0.997 and − 0.996, respectively. For the sensible heat transport in the lower troposphere (Fig. 4c,e), heat transport is mostly directed away from the Arctic due to a weaker mean heat influx in the Eastern Hemisphere than heat outflux in the Western Hemisphere. Thus, in the lower troposphere, the direction of heat fluxes in the hemispheres is flipped. The correlation coefficients between the heat transports in the hemispheres for the unsmoothed and smoothed time series (Fig. 4c) are − 0.88 and − 0.80, respectively.

For the latent heat transport in the entire troposphere (Fig. 4b,f), heat transport is mostly directed into the Arctic due to a combined effect of mean positive heat influxes in the Eastern and Western Hemispheres. This is also confirmed by a large area of intersection for the interquartile ranges in Fig. 4b and by significantly higher absolute values for the 75th percentiles than for the 25th percentiles. However, the clearly seen anti-phase pattern is preserved, and the correlation coefficients between the heat transports in the hemispheres for the unsmoothed and smoothed time series (Fig. 4b) are − 0.82 and − 0.77, respectively. For the latent heat transport in the lower troposphere (Fig. 4d,f), heat transport is also mostly directed into the Arctic, but due to a stronger mean heat influx in the Eastern Hemisphere than heat outflux in the Western Hemisphere. The correlation coefficients between the heat transports in the hemispheres for the unsmoothed and smoothed time series (Fig. 4d) are − 0.66 and − 0.54, respectively.

The internal energy budgets defined by the sums of sensible and latent heat fluxes between the hemispheres are − 0.65 ± 0.09 PW for the lower troposphere 1000–800 hPa and 4.28 ± 0.18 PW in the entire troposphere 1000–100 hPa. In both cases, the overall sign is defined by the several orders of magnitude higher absolute values of sensible heat flux than of latent heat flux. The mean values and corresponding uncertainties for the intermediate cases are shown in the Table 1.

**Table 1:** Mean heat fluxes of sensible heat transport (SHT) and latent heat transport (LHT) for the Eastern Hemisphere (EH) and Western Hemisphere (WH).

	SHT, PW (10^15^ W)		LHT, TW (10^12^ W)	
	EH	WH	EH	WH
1000–800 hPa	1.35 ± 0.19	− 2.02 ± 0.17	19.98 ± 3.83	− 13.16 ± 2.64
1000–100 hPa	− 11.14 ± 2.11	15.38 ± 2.17	18.01 ± 7.15	24.75 ± 6.40
Net flux (1000–800 hPa)	− 0.66 ± 0.09		6.81 ± 2.89	
Net flux (1000–100 hPa)	4.24 ± 0.18		42.75 ± 4.12	

### Experimental testing of the robustness of the identified dipole pattern for the sensible and latent heat transports in the entire troposphere

**Figure 5:**
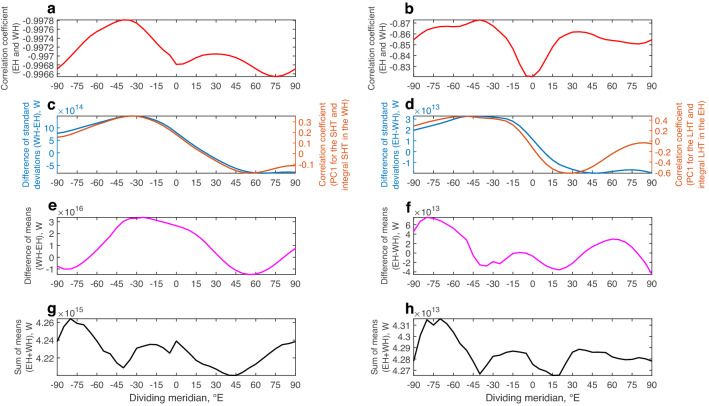
Sensitivity experiments for the dipole pattern in the integral sensible (SHT (W), left) and latent (LHT (W), right) heat transports in the entire troposphere (1000–100 hPa) for the Eastern and Western Hemispheres (EH and WH). (**a**) Anti-phase pattern between the EH and WH for the SHT. (**b**) Anti-phase pattern between the EH and WH for the LHT. (**c**) Difference of standard deviations for the SHT (WH – EH) along with the correlation coefficients between the first principal component time series (PC1) for the SHT and integral SHT in the WH. (**d**) Difference of standard deviations for the LHT (EH – WH) along with the correlation coefficients between the first principal component time series (PC1) for the LHT and integral LHT in the EH. (**e**) Difference of means (WH − EH) for the SHT. (**f**) Difference of means (EH – WH) for the LHT. (**g**) Sum of means (EH + WH) for the SHT. (**h**) Sum of means (EH + WH) for the LHT. The Matrix Laboratory (MATLAB) with version R2017b (https://www.mathworks.com/) was used to generate this figure.

To investigate the uniqueness of the identified hemispheric patterns, sensitivity experiments were carried out (see “Methods” for the detailed description). The results are presented in Fig. 5.

As seen, the anti-phase pattern in the heat transport components is not a unique feature for the geographical definitions of the Eastern and Western Hemispheres. Moreover, the highest anti-correlations are for the dividing meridians around 35°–40°W although numerical changes for the sensible heat transport are almost negligible and quite low for the latent heat transport (Fig. 5a,b). Such robustness of the anti-phase pattern between the hemispheres, regardless of the dividing meridian, might be related to the climatological meridional wind velocity fields (Fig. 1). Namely, for any large-scale regional division into two parts, there will be two wind anomalies for each hemisphere. In turn, one of the anomalies in the Eastern Hemisphere will always dominate over the other, whereas in the Western Hemisphere, the situation is the same, but with a different sign of the dominating anomaly. Therefore, two dominating anomalies of different signs give rise to the anti-phase pattern between the hemispheres.

The dominance of variability in the Western Hemisphere over the Eastern Hemisphere for the sensible heat transport is confirmed (Fig. 5c) because the highest differences of standard deviations are of positive sign. They achieve their maximum values for the dividing meridians around 30°–40°W although the prime meridian is also a good indicator of the difference in the variability between the hemispheres. The correlation function, representing the interrelation between the first principal component time series for the sensible heat transport and integral sensible heat transport in the Western Hemisphere, is nearly identical to the curve for the differences of standard deviations between the Western and Eastern Hemispheres. The correlation coefficient between the two curves is 0.99. These results confirm the correctness and physical significance of the first EOF for the sensible heat transport (Fig. 2a). The dominance of variability in the Eastern Hemisphere over the Western Hemisphere for the latent heat transport is also confirmed (Fig. 5d). Compared to Fig. 5c, the best dividing meridians are slightly shifted westwards, and the correlation coefficient between the curves is 0.88 which is lower than in Fig. 5c, but still very high. Thus, the correctness and physical significance of the first EOF for the latent heat transport (Fig. 2b) is also confirmed.

The distribution of the difference of means for the sensible heat transport in Fig. 5e resembles the curves in Fig. 5c, with the maximum differences also around the dividing meridian 30°W. The correlation coefficients between the magenta curve in Fig. 5e and the blue and orange curves in Fig. 5c are 0.68 and 0.71, respectively. These results confirm that for the sensible heat transport, the best dividing meridian into two hemispheres may be around 30°–40°W. However, the prime meridian is not significantly worse. The distribution for the latent heat transport in Fig. 5f is much more complicated, which might be related to the fact that for this heat transport component, there is a combined effect of mean positive heat influxes in the Eastern and Western Hemispheres (Fig. 4b).

The distributions of the sums of means (Fig. 5g,h) show quite similar behavior, with a correlation coefficient of 0.60. Moreover, for each heat transport component, the sign is always positive. This indicates a low sensitivity for the sums of heat transports with respect to the dividing meridian.

Nevertheless, the average vertical distributions of heat transports in the hemispheres for the dividing meridian of 35°W (Supplementary Fig. S3) show the anti-phase pattern for the latent heat transport worse than for the prime meridian in Fig. 3. However, the integral sensible and latent heat transports (Supplementary Fig. S4) confirm the strong anti-phase pattern although its visual perception might be better for the prime meridian in Fig. 4.

## Discussion and conclusions

This study reports the discovery of a dipole pattern of the meridional sensible and latent heat transports into the Arctic between the Eastern and Western Hemispheres. This pattern is pronounced in the different variability and anti-phase feature between the hemispheres. The physical significance of the large-scale hemispheric division obtained from the EOF analysis across the zonal section at 70°N has been confirmed by the analysis of time-altitude diagrams in the areas longitudinally averaged for the Eastern Hemisphere and Western Hemisphere, fully integrated sensible and latent heat transports, and the sums of these transports. An additional confirmation has been obtained from the sensitivity experiments with respect to the dividing meridian. The latent heat transport is a major contributor to the heat excess into the Arctic. For this component, the net heat flux is positive both in the lower and in the entire troposphere.

What might explain the dominance of latent heat transport in the Eastern Hemisphere and sensible heat transport in the Western Hemisphere? This requires further in-depth studies. However, we think the exact clues are hidden in the following conceptual explanation. Regarding the dominance of latent heat transport in the Eastern Hemisphere, first, the extratropical cyclones, originating in the North Atlantic, carry moisture to the northeast, thus affecting the Arctic in the western and central parts of the Eastern Hemisphere^20–22^. Second, the cyclone tracks from the North Pacific are predominantly directed northwest^23^, thus affecting the Arctic in the far eastern area of the Eastern Hemisphere. Third, the Siberian atmospheric rivers are responsible for the dominance of latent heat transport in the central and eastern parts of the Eastern Hemisphere^24^. The three different processes work simultaneously and give rise to the large-scale convergence of moisture transport into the Arctic in the Eastern Hemisphere. According to the results of our study, the dominance in the variability of sensible heat transport in the Western Hemisphere is over the isobaric surfaces 1000–100 hPa. This might be related to the trend in the cold air outbreaks, which has a pronounced negative sign in the Western Hemisphere according to the ERA5 reanalysis; at the same time, the frequency and magnitude of cold air outbreaks in the Eastern Hemisphere have not changed significantly since 1979^25^.

## Methods

### Data

In this study, we used the ERA5 climate reanalysis at monthly temporal resolution for the period from January 1950 to December 2019. The reanalysis for the period 1950–1978 is only the preliminary version and is subject to changes yet due to unrealistically strong tropical cyclones, but the present era from 1979 is fully completed^26^. As our focus is on the high-latitude processes, we assume that the current issues for 1950–1978 should not affect the results presented here. It is important to note that the entire period 1950–2019 covers known cooling and warming periods of the present climate^27–29^. The following variables were downloaded:

1. Monthly mean meridional component of wind velocity (m s^−1^) on the 27 isobaric surfaces 1000–100 hPa in the area 60°N–80°N with a horizontal resolution 1° × 1° and vertical resolution 25–50 hPa (each available isobaric surface has been used). The highest spatial resolution 0.25° × 0.25° was not used, as it is not critical for this part of study and allows saving computational resources.
2. Monthly mean meridional component of wind velocity (m s^−1^) on the 27 isobaric surfaces 1000–100 hPa at the latitude 70°N with a horizontal resolution 0.25° × 0.25° and vertical resolution 25–50 hPa.
3. Monthly mean air temperature (K) on the 27 isobaric surfaces 1000–100 hPa at the latitude 70°N with a horizontal resolution 0.25° × 0.25° and vertical resolution 25–50 hPa.
4. Monthly mean specific humidity (kg kg^−1^) on the 27 isobaric surfaces 1000–100 hPa at the latitude 70°N with a horizontal resolution 0.25° × 0.25° and vertical resolution 25–50 hPa.
5. Monthly mean geopotential (m^2^s^−2^) on the 27 isobaric surfaces 1000–100 hPa at the latitude 70°N with a horizontal resolution 0.25° × 0.25° and vertical resolution 25–50 hPa. These data were transformed to the geopotential heights (m) after dividing by the gravitational acceleration (9.80665 m s^−2^).

### Investigation of the monthly northward wind fields around the parallel 70°N

Here, we found the mean meridional component of wind velocity at every grid point for the region 60°N–80°N in the three atmospheric layers (1000–800 hPa, 775–400 hPa and 350–100 hPa), which is nine isobaric surfaces per layer. The monthly values were first averaged over the isobaric surfaces, and then temporal means were found for 1950–2019.

### Calculation of the monthly meridional sensible and latent heat transport (SHT and LHT) components through the latitude 70°N and their decomposition into the empirical orthogonal functions (EOFs)

The sensible (SHT) and the latent (LHT) heat transports (W m^−2^) were computed as follows:

1 $$SHT_{{l,p,t\left( {70^\circ N} \right)}} = C_{p} \rho T_{l,p,t} V_{l,p,t}$$

2 $$LHT_{l,p,t(70^\circ N)} = L_{v} \rho Q_{l,p,t} V_{l,p,t} ,$$

where C_p_ is the mean specific heat capacity of air at constant pressure equal to 1005 J kg^−1^ K^−1^, L_v_ is the mean latent heat of vaporization equal to 2.5 × 10^6^ J kg^−1^, l is the longitude, p is the isobaric surface, t is time, ρ is the mean air density equal to 1.3 kg m^−3^, T is the monthly mean air temperature (K), Q is the monthly mean specific humidity (kg kg^−1^), V is the monthly mean northward wind velocity (m s^−1^).

This method was used in Alekseev et al.^11^, but that study had a regional focus in the Atlantic-European part. The Eqs. (1) and (2) represent the instantaneous meridional fluxes because they are estimated for every longitude and isobaric surface at a given time. This approach provides much more detailed structure on the individual heat transport components than previous studies where these were computed from spatial (zonal and vertical) and temporal means^3^. To investigate large-scale features of heat fluxes, we decomposed the sensible and latent heat transports across 70°N (functions of longitude, isobaric surface, and time) into the empirical orthogonal functions (EOFs) following the techniques described in Greene et al.^30^. Therefore, the monthly time series were both detrended and deseasoned prior to the EOF analysis.

### Investigation of the vertical distributions of the annual meridional sensible and latent heat transport (SHT and LHT) components through the latitude 70°N

The longitudinal averaging was done for the Eastern and Western Hemispheres (0°–179.75°E and 180°–0.25°W, respectively) because the EOF analysis suggested different variability in these regions. The noisy monthly time series were averaged for every year to highlight the vertical structure of heat fluxes in the entire troposphere 1000–100 hPa by means of time-altitude diagrams. The standard deviations were analyzed along the temporal and vertical dimensions (over the years and isobaric surfaces) to analyze how the sensible and latent heat transports are controlled in the Eastern and Western Hemispheres. The annual means were used for this part of study only. In all other cases, monthly time series were analyzed.

### Integral sensible and latent heat transports (SHT and LHT) in the lower and entire troposphere and their net fluxes through the Arctic gate

The monthly times series of sensible and latent heat transports were further integrated (SHT_int._ and LHT_int._, W) in both hemispheres for two atmospheric layers previously identified by the mean directions of fluxes: 1000–800 hPa and 1000–100 hPa as written in the Eqs. (3) and (4):

3 $$SHT_{{{\text{int}} .}} = \int\limits_{{p_{1} }}^{{p_{2} }} {\int\limits_{{l_{1} }}^{{l_{2} }} {(SHT_{l,p,t(70^\circ N)} )dldp} }$$

4 $$LHT_{{{\text{int}} .}} = \int\limits_{{p_{1} }}^{{p_{2} }} {\int\limits_{{l_{1} }}^{{l_{2} }} {(LHT_{l,p,t(70^\circ N)} )dldp} },$$

where p_1_ and p_2_ correspond to the layers 1000–800 hPa and 1000–100 hPa, l_1_ and l_2_ mark the limits of either the Eastern Hemisphere (0°–179.75°E) or the Western Hemisphere (180°–0.25°W), the horizonal integration step dl = 0.25°, the vertical integration step dp = 25 or 50 hPa in accordance with the data on geopotential heights (m) at each grid point corresponding to the isobaric surfaces. Numerical integration was carried out by the trapezoidal method.

In the time series obtained the mean values were calculated for every case, and the confidence interval for the uncertainties of the means was estimated based on the *t*-distribution for the 5% significance level^31^. Subsequently, the net sensible and latent heat transports were found by adding the time series in the Eastern and Western Hemispheres for every atmospheric layer. The obtained time series can be interpreted as the net heat fluxes for the given latitude (70°N). The internal energy budgets for the lower and entire troposphere were calculated by adding the sensible and latent heat fluxes in the hemispheres. Plotting of the monthly integral heat fluxes was done after applying the loess smoothing with a span of 5% of the values to smooth out high-frequency data and highlight the anti-phase pattern between the hemispheres. The interquartile ranges (differences between 75th and 25th percentiles) for each pair of the time series were computed for the unsmoothed monthly time series, and the corresponding shaded areas have been added to the curves. The Pearson correlation coefficients were computed for both unsmoothed and filtered time series displayed in the figure.

### Sensitivity experiments for the dipole pattern of sensible and latent heat transports in the entire troposphere

In this section, we calculated the integral sensible and latent heat transports in the entire troposphere 1000–100 hPa for the Eastern and Western Hemispheres with different dividing meridians. We did that for the range from 90°W to 90°E with a step of 5°. For example, for the dividing meridian of 5°E, the Eastern Hemisphere spans the range from 5°E to 175.25°W (from left to right), and the Western Hemisphere spans the range from 175°W to 4.75°E (from left to right). Thus, the number of longitudes in each hemisphere is always equal. For the time series obtained, the correlation coefficients were computed between the hemispheres to test the robustness of the anti-phase pattern. In order to check the EOF patterns, the differences of standard deviations were computed between the dominant hemisphere and non-dominant hemisphere for the corresponding heat transport components. These curves were plotted together with the correlations between the first principal component time series for the sensible and latent heat transports and integral heat transports for the respective dominant hemispheres (Western Hemisphere for the sensible heat transport and Eastern Hemisphere for the latent heat transport). In addition, the sensitivities for the difference and sum of means between the hemispheres were tested. Every curve was plotted as a function of the dividing meridian. All numerical estimations were obtained for the unsmoothed monthly time series.

## Supplementary Information

Supplementary Figures.

## Data Availability

The ERA5 reanalysis data used in this study are publicly available from the Copernicus Climate Change Service: https://climate.copernicus.eu/climate-reanalysis.

## Code availability

The MATLAB codes used for the calculations and creation of Figures discussed in this work have been archived and are available on request from the corresponding author, M.M.L. (m.m.latonin@spbu.ru).
