# Supplementary material for: Dipole pattern of meridional atmospheric internal energy transport across the Arctic gate
Source: Sci Rep. 2022 Feb 11;12:2363. doi: 10.1038/s41598-022-06371-9 (PMC8837744; doi:10.1038/s41598-022-06371-9)
Supplement: Supplementary file 1 — Supplementary Figures. [file 41598_2022_6371_MOESM1_ESM.pdf]

Dipole pattern of meridional atmospheric internal energy transport across the Arctic gate

Mikhail M. Latonin[a,b,*], Leonid P. Bobylev[b], Igor L. Bashmachnikov[a,b], Richard Davy[c]

[a]*Saint Petersburg State University, Universitetskaya Emb. 7–9, 199034 Saint Petersburg, Russia; i.bashmachnikov@spbu.ru*

[b]*Nansen International Environmental and Remote Sensing Centre, 14th Line 7, Office 49, Vasilievsky Island, 199034 Saint Petersburg, Russia; leonid.bobylev@niersc.spb.ru*

[c]*Nansen Environmental and Remote Sensing Center, and Bjerknes Center for Climate Research, Jahnebakken 3, N-5007 Bergen, Norway; richard.davy@nersc.no*

*Corresponding author.

E-mail address: m.m.latonin@spbu.ru

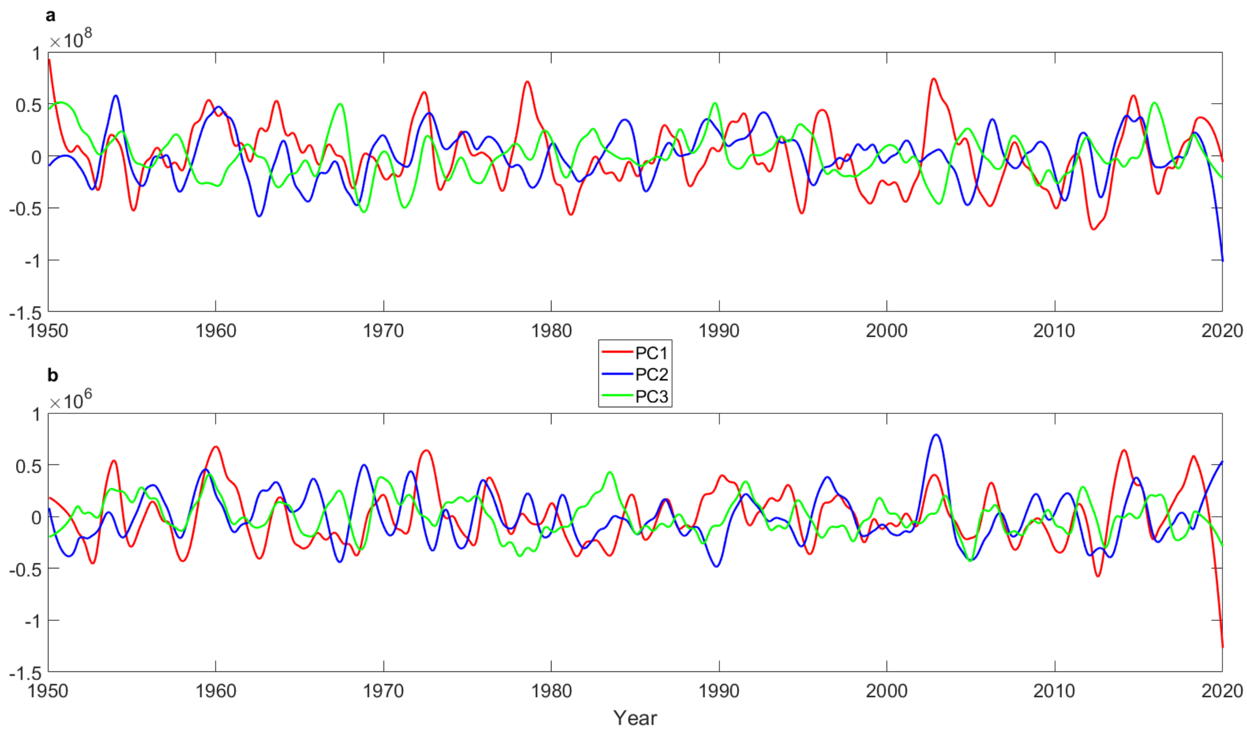

Fig. S1 Smoothed principal component (PC) time series for the first three empirical orthogonal functions (EOFs) of the sensible and latent heat transport components (SHT and LHT). a, PC time series for the SHT. **b**, PC time series for the LHT. The loess smoothing was performed with a span of 5% of the values. The Matrix Laboratory (MATLAB) with version R2017b (https://www.mathworks.com/) was used to generate this Figure.

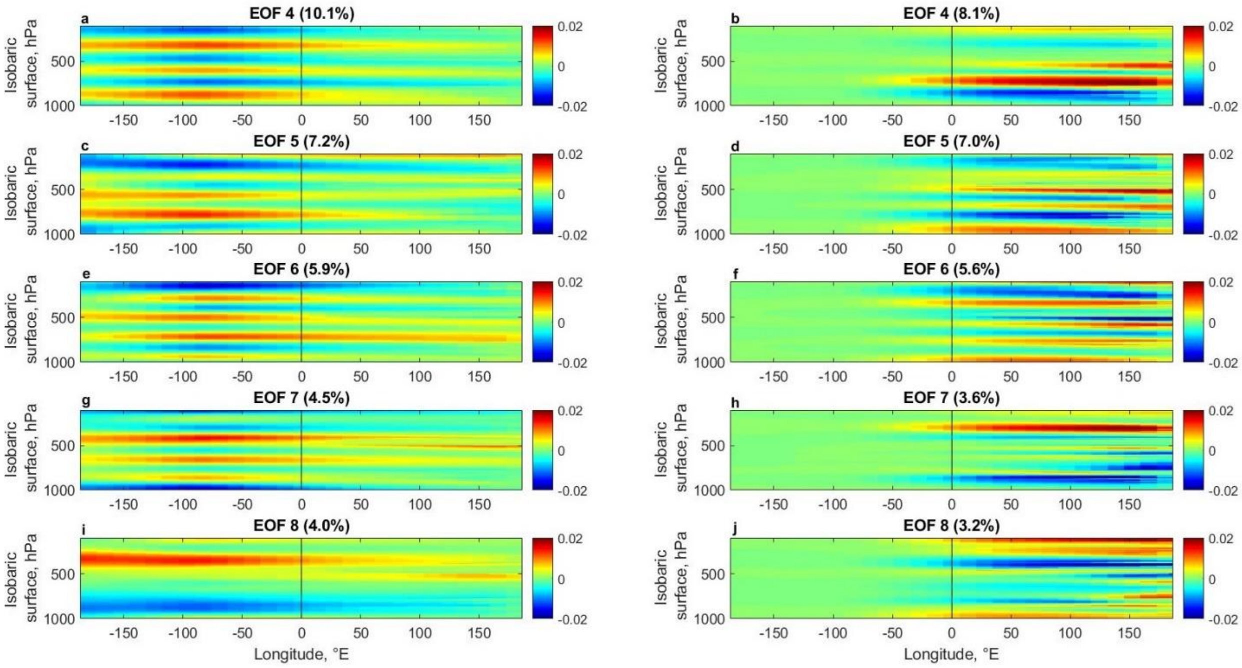

Fig. S2 Empirical orthogonal functions (EOFs) of the sensible (left) and latent (right) heat transport components (SHT and LHT). a, Fourth EOF for the SHT (*10.1%*). **b**, Fourth EOF for the LHT (*8.1%*). **c**, Fifth EOF for the SHT (*7.2%*). **d**, Fifth EOF for the LHT (*7.0%*). **e**, Sixth EOF for the SHT (*5.9%*). **f**, Sixth EOF for the LHT (*5.6%*). **g,** Seventh EOF for the SHT (*4.5%*). **h,** Seventh EOF for the LHT (*3.6%*). **i,** Eighth EOF for the SHT (*4.0%*). **j,** Eighth EOF for the LHT (*3.2%*). The reference lines are drawn at the prime meridian 0°. The values in the parentheses are the fractions of variance explained by each mode. The Matrix Laboratory (MATLAB) with version R2017b (https://www.mathworks.com/) was used to generate this Figure.

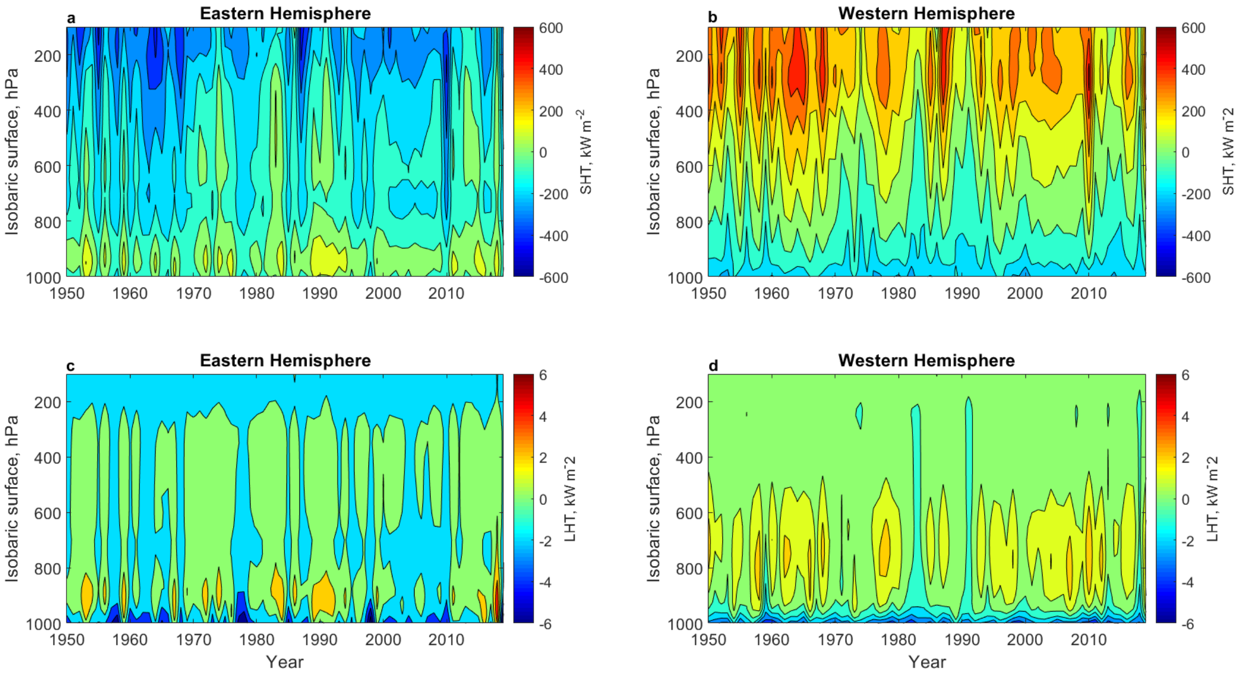

Fig. S3 Vertical cross-section of heat transport components (kW m^{-2}), their interannual variability and the anti-phase pattern between the hemispheres. a, Annual average sensible heat transport in the Eastern Hemisphere (35°W–144.75°E). **b**, Annual average sensible heat transport in the Western Hemisphere (145°E–35.25°W). **c**, Annual average latent heat transport in the Eastern Hemisphere (35°W–144.75°E). **d**, Annual average latent heat transport in the Western Hemisphere (145°E–35.25°W). The positive values indicate northward heat transport. The Matrix Laboratory (MATLAB) with version R2017b (https://www.mathworks.com/) was used to generate this Figure.

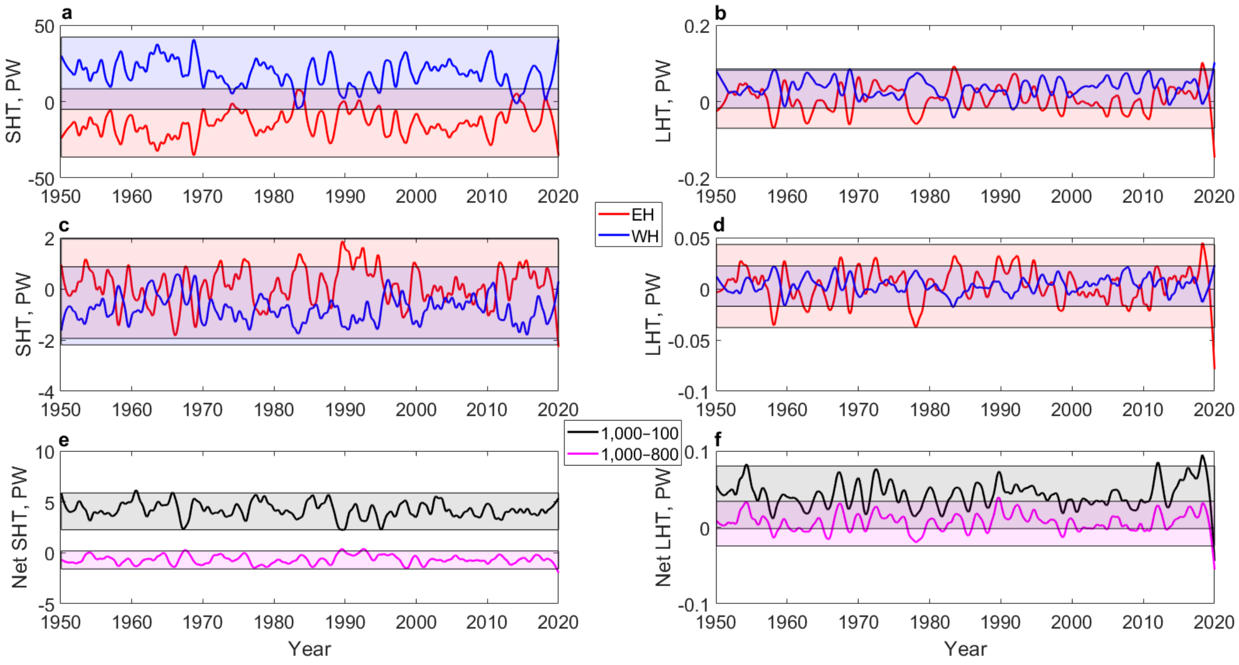

Fig. S4 Smoothed time series of integral sensible and latent heat transport (SHT and LHT) components (Petawatt (PW)) in the lower and entire troposphere for the Eastern (35°W–144.75°E) and Western (145°E–35.25°W) Hemispheres (EH and WH) and the net fluxes. a, 1000–100 hPa integral SHT in the EH and WH. **b**, 1000–100 hPa integral LHT in the EH and WH. **c**, 1000–800 hPa integral SHT in the EH and WH. **d**, 1000–800 hPa integral LHT in the EH and WH. **e**, Net fluxes of SHT (EH + WH). **f**, Net fluxes of LHT (EH + WH). The lighter shaded areas indicate the interquartile ranges (differences between 75th and 25th percentiles) for the unsmoothed monthly time series, and the darker shaded areas show the intersections of the interquartile ranges for each pair of the time series. The loess smoothing was performed with a span of 5% of the values. The Matrix Laboratory (MATLAB) with version R2017b (https://www.mathworks.com/) was used to generate this Figure.
